# Supplementary material for: Age-associated mortality is partially mediated by TERT promoter mutation status in differentiated thyroid carcinoma
Source: PLoS One. 2023 Nov 10;18(11):e0294145. doi: 10.1371/journal.pone.0294145 (PMC10637683; doi:10.1371/journal.pone.0294145)
Supplement: S1 Table — (DOCX) [file pone.0294145.s001.docx]

|  | ***N*** | **10-year survival rate (%)** | **Univariate Cox models** | | | |
| --- | --- | --- | --- | --- | --- | --- |
| **Variables** |  |  | **Hazard ratio** | **95% lower** | **95% upper** | ***P* value** |
| Sex |  |  |  |  |  |  |
| Female | 329 | 95.1 | 1.00 (reference) | | | 0.167 |
| Male | 64 | 92.2 | 1.83 | 0.78 | 4.34 |  |
| Age at diagnosis (years) |  |  |  |  |  |  |
| <55 | 319 | 97.5 | 1.00 (reference) | | | <0.001 |
| ≥55 | 74 | 82.4 | 11.34 | 4.96 | 25.93 |  |
| *TERT* promoter mutations |  |  |  |  |  |  |
| WT | 350 | 98.0 | 1.00 (reference) | | | <0.001 |
| Mutation | 43 | 67.4 | 24.15 | 10.56 | 55.25 |  |
| *BRAF* V600E mutation |  |  |  |  |  |  |
| WT | 117 | 92.3 | 1.00 (reference) | | | 0.331 |
| Mutation | 199 | 97.0 | 0.64 | 0.26 | 1.57 |  |
| Lymph node metastasis |  |  |  |  |  |  |
| Absent | 199 | 94.0 | 1.00 (reference) | | | 0.964 |
| Present | 193 | 95.3 | 1.02 | 0.47 | 2.20 |  |
| Extrathyroidal extension |  |  |  |  |  |  |
| Absent | 352 | 96.0 | 1.00 (reference) | | | <0.001 |
| Present | 41 | 82.9 | 4.78 | 2.14 | 10.64 |  |
| Distant metastasis |  |  |  |  |  |  |
| Absent | 370 | 96.8 | 1.00 (reference) | | | <0.001 |
| Present | 23 | 60.9 | 10.85 | 4.86 | 24.22 |  |
| Tumor size |  |  |  |  |  |  |
| <2.0 cm | 45 | 95.6 | 1.00 (reference) | | | 0.014 |
| 2.0-4.0 cm | 293 | 95.9 | 1.24 | 0.28 | 5.38 | 0.776 |
| >4.0 cm | 55 | 87.3 | 3.96 | 0.86 | 18.34 | 0.078 |
| RAI total dose |  |  |  |  |  |  |
| Per 1 mCi | 393 |  | 1.0033 | 1.0024 | 1.0043 | <0.001 |

**S1 Table**  The univariable analysis of the association between clinicopathological variables and cancer-specific survival (CSS) in patients with differentiated thyroid cancer

Abbreviations: *TERT*, telomerase reverse transcriptase; RAI, radioactive iodine.
