# Supplementary material for: Age-associated mortality is partially mediated by TERT promoter mutation status in differentiated thyroid carcinoma
Source: PLoS One. 2023 Nov 10;18(11):e0294145. doi: 10.1371/journal.pone.0294145 (PMC10637683; doi:10.1371/journal.pone.0294145)
Supplement: S2 Table — (DOCX) [file pone.0294145.s002.docx]

**S2 Table**  The univariable analysis of the association between clinicopathological variables and cancer-specific survival (CSS) in patients with papillary thyroid cancer

|  | ***N*** | **10-year survival rate (%)** | **Univariate Cox models** | | | |
| --- | --- | --- | --- | --- | --- | --- |
| **Variables** |  |  | **Hazard ratio** | **95% lower** | **95% upper** | ***P* value** |
| Sex |  |  |  |  |  |  |
| Female | 276 | 97.5 | 1.00 (reference) | | | 0.026 |
| Male | 51 | 92.2 | 3.09 | 1.14 | 8.36 |  |
| Age at diagnosis (years) |  |  |  |  |  |  |
| <55 | 265 | 98.1 | 1.00 (reference) | | | <0.001 |
| ≥55 | 62 | 90.3 | 11.00 | 3.87 | 31.23 |  |
| *TERT* promoter mutations |  |  |  |  |  |  |
| WT | 295 | 99.0 | 1.00 (reference) | | | <0.001 |
| Mutation | 32 | 75.0 | 36.27 | 11.81 | 111.42 |  |
| *BRAF* V600E mutation |  |  |  |  |  |  |
| WT | 65 | 98.5 | 1.00 (reference) | | | 0.253 |
| Mutation | 199 | 97.0 | 3.31 | 0.42 | 25.89 |  |
| Lymph node metastasis |  |  |  |  |  |  |
| Absent | 135 | 97.0 | 1.00 (reference) | | | 0.410 |
| Present | 191 | 96.3 | 1.56 | 0.54 | 4.49 |  |
| Extrathyroidal extension |  |  |  |  |  |  |
| Absent | 290 | 97.6 | 1.00 (reference) | | | 0.002 |
| Present | 37 | 89.2 | 4.67 | 1.73 | 12.65 |  |
| Distant metastasis |  |  |  |  |  |  |
| Absent | 313 | 97.4 | 1.00 (reference) | | | 0.007 |
| Present | 14 | 78.6 | 5.59 | 1.61 | 19.47 |  |
| Tumor size |  |  |  |  |  |  |
| <2.0 cm | 35 | 97.1 | 1.00 (reference) | | | 0.087 |
| 2.0-4.0 cm | 253 | 97.2 | 1.53 | 0.20 | 11.84 | 0.685 |
| >4.0 cm | 39 | 92.3 | 4.70 | 0.55 | 40.19 | 0.158 |
| RAI total dose |  |  |  |  |  |  |
| Per 1 mCi | 327 |  | 1.00308 | 1.00174 | 1.00442 | <0.001 |

Abbreviations: *TERT*, telomerase reverse transcriptase; RAI, radioactive iodine.
