# Supplementary material for: Age-associated mortality is partially mediated by TERT promoter mutation status in differentiated thyroid carcinoma
Source: PLoS One. 2023 Nov 10;18(11):e0294145. doi: 10.1371/journal.pone.0294145 (PMC10637683; doi:10.1371/journal.pone.0294145)
Supplement: Attachment — Submitted filename: Response to reviewers.docx [file pone.0294145.s003.docx]

**[Response to Reviewers’ comments]**

**Manuscript Number: PONE-D-23-13935**

**Article Type: Original article**

**Title: Age-associated mortality is partially mediated by TERT promoter mutation status in differentiated thyroid carcinoma**

**[Additional Editor Comments]**

Some potential articles in this field are not cited and are worth to cite the following articles as they contributed to the development of this field (PMID: 23766237; PMID: 25024077; PMID: 26354077; PMID: 26711586; PMID: 26902827).

**🡪 Response**: We appreciate the editor’s introduction of great articles that we overlooked for citation. We have added suitable articles to the references (Ref 7–9, 46–47).

**[Reviewer #1]**

**[Major comments]**

1) Many of the figure legends are missing or have lack of the information. For example, figure 2, 3, 4, and 5 have only title and there are no figure legend which can explain about detail of the figure. It would be better to have abbreviation, statistic information, explanation about each subfigure (such as A, B, and C), etc. in the legend. Especially, in the case of dual axis graph, like figure 4, detailed figure legend should be needed to prevent confusion.

**🡪 Response:** Thank you for the valuable comment. We have enhanced the figure legends by adding detailed information.

- **[Revised]** Line 198-203, 239–247, 258–266, 311–316, 344–347.

2) In figure 2 and related manuscript, the authors addressed that “The mortality rates were low in all patients with DTC and in the WT-TERT group before the age of 55 years compared with the high mortality rate in the M-TERT group”. To conclude more precisely, the author need to add statistical analysis with comparison between “all vs M-TERT” and “WTTERT vs M-TERT” and provide information in the figure 2.

**🡪 Response:** According to the comment, we performed Cochran-Mantel-Haenszel (CMH) test to compare the mortality rate between WT-*TERT* and M-*TERT* groups, and the mortality rate was higher in M-*TERT* than WT-*TERT* (*p*=0.005). We added this result in the revised manuscript regarding Fig 2.

As for “All vs. M-*TERT*”, concerning that “All” encompasses “M-*TERT*”, the phrase about “All vs. M-*TERT*” was removed from the original text, although M-*TERT* was significantly associated with shorter cancer-specific survival compared to both all patients with DTC and WT-*TERT* (log-rank test *p*<0.001).

- **[Revised]** Line 147–149, 231–233

3) Based on the mutation analysis, the authors could see two different type of promoter mutations, C228T and C250T. Have there been any previous studies conducted on which mutation is a more risk factor or more frequent in diseases other than DTC (for example, glioblastoma multiforme, GBM)? If so, it would be great to do more discussion in addition to line 429-434.

**🡪 Response:** Thank you for the valuable comment. We added a paragraph discussing previous studies on the difference between C228T and C250T as prognostic factors in cancers other than thyroid cancer.

- **[Revised]** Line 375–386

**[Minor comments]**

1) In Table 4 and related manuscript, the authors showing result of mediation analysis. Even though they mentioned in the introduction part (line 272) that “natural direct effect (NDE), a synonym for PDE, and the natural indirect effect (NIE), a synonym for TIE”, it would be great to understand for the reader using unified term. In addition, please add abbreviation as well in the table; for example, Total effect (TE), Pure direct effect (PDE), etc. It makes the easier to follow.

**🡪 Response:** Thank you for the opinion. We removed the potentially confusing terms (NDE and NIE) and unified the terminology. Also, we added abbreviations in the Table 4.

- **[Revised]** Line 333–334 and 1^st^ column of Table 4

**[Reviewer #2]**

1) Most of the patients in this cohort has papillary thyroid cancer, which occurs more frequently in women. It seems that gender is an unneglectable factor associated to the differentiated thyroid carcinoma. In table 1 and S1, the authors listed the numbers of females and males of the patient cohort and the number of patients with TERT promoter mutations. However, they did not specify the number of female and male in the M-TERT group. Is there a difference in the incidence of TERT promoter mutations between males and females?

- **Response:** In DTC, the incidence of *TERT* promoter mutation were 10.9% (36/329) in female, and 10.9% (7/64) in male, respectively (*p*=1.000). In PTC, the incidence of the mutation were 9.1% (25/276) in female, and 13.7% (7/51) in male, respectively (*p*=0.214).

Moreover, what is the incidence of TERT promoter mutations in different age groups?

- **Response:** The incidence of *TERT* promoter mutation in each age groups are 1.8% (4/224) in age<45, 11.6% (11/95) in age 45–54, 23.3% (10/43) in age 55–64, and 58.1% (18/31) in age≥65, respectively.

As presented in figures 2, 3B, and 3E, there is a clear linear association between mortality rate and age in patients with WT-TERT promoters, whereas this linear association is not found in patients with mutated TERT promoters. The mortality rate is lower in the 55-64y age group as compared to that in the 45-54y age group in patients with mutated TERT promoters**.** Have the authors examined the mortality rate separately in males and females in different age groups?

**🡪 Response:** As shown in the additional Fig 1 and 2, mortality rate increased with age in total patients with DTC in both male and female subgroups (black curves). In M-*TERT*, the mortality rate decreased with age in males (additional Fig 1), and the mortality rate showed a “Z shape” in females (additional Fig 2) (*p* for trend; 0.147 in males and 0.054 in females) (red curves). Of note, the graph of mortality rate in total M-*TERT* is similar to that of female M-*TERT*. This may be due to the dominance of females over males, which is approximately 5:1 in the study cohort. In WT-*TERT*, the mortality rate increased with age in the male subgroup (*p* for trend=0.002), whereas that of the female subgroup was highest in the 55-64 age group, and lower in the > 65 age group than in the 55-64 age group (*p* for trend=0.0001) (yellow curves).

We appreciate the reviewer’s suggestion for advanced analyses of age- and gender-specific mortality rates. Mortality patterns varied by gender in both WT-*TERT* and M-*TERT*, as noted above. However, we regret to inform the reviewer that the number of deaths in each age and gender subgroup was too small, particularly with only seven total deaths in the entire male group, to allow meaningful comparative analysis of mortality by age and gender subgroups.

Females go through menopause between 45 and 55, and there is a decline of estradiol production during this period. It has been reported that 17beta-estradiol significantly increased telomerase activity in human endothelial cells (Grasselli et al., Circ Res. 2008 Jul 3;103(1):34-42), therefore, it is worthwhile and crucial to explore the interaction of age, gender and TERT promoter mutations in this cohort of patients.

**🡪 Response:**

The authors understand the reviewer’s point regarding the need to investigate the effect of menopause and changes in E2 levels on TERT activity in female patients. Despite the intriguing perspective, there was no obvious pattern observed in mortality rates around the age of 45-54 in the female group, especially within the WT-*TERT* where the influence of *TERT* promoter mutation was excluded, suggesting that E2-dependent induction of hTERT would be a cell type specific event requiring NO production. Thus, a better understanding of the TERT activity based on estradiol levels may be pursued in the field of thyroid cancer. We have mentioned this point in the Discussion section.

- **[Revised]** Line 406–410.

2) To make it clear and easier for reading, please put subtitles to each graph to indicate the group of patients.

**🡪 Response:** Thank you for the valuable comment. We have enhanced the figure legends by adding detailed information explaining each graph.

- **[Revised]** Line 198-203, 239–247, 258–266, 311–316, 344–347.

**[Reviewer #3]**

Aging and TERT promoter mutations have been previously shown as prognostic factors in Differentiated Thyroid Carcinoma (Tae Hyuk Kim et al., 2016). In this study, authors used their previous Differentiated Thyroid Carcinoma (DTC) cohort to elucidate the interaction of aging and TERT promoter mutations. They found that the effect of aging on mortality of mutant TERT patients were not significant, while aging caused higher mortality rate in Wild type TERT patients. In addition, Heo J et al., found that TERT promoter mutation contributes to 36% of age-related mortality rate in this DTC cohort. Next, authors concluded that TERT promoter mutations should be considered in a stage classification of DTC. Although these finding are interesting observations, but the conclusion of this study is similar to their previous study (Tae Hyuk Kim et al., 2016).

**🡪 Response:** Thank you for the comment, and we agree with the reviewer’s opinion that conclusion of this study is similar to previous studies. What is different from previous studies is that this study found *TERT* promoter mutation contributes partially to the prognostic role of age at diagnosis in DTC. To emphasize this distinguishing factor, we partially modified the manuscript mentioning the need for further research on how to incorporate *TERT* with age into a new staging system (for example, different age cutoffs based on *TERT* promoter mutation status).

- **[Revised]** Line 414-421
