# Supplementary material for: Age-associated mortality is partially mediated by TERT promoter mutation status in differentiated thyroid carcinoma
Source: PLoS One. 2023 Nov 10;18(11):e0294145. doi: 10.1371/journal.pone.0294145 (PMC10637683; doi:10.1371/journal.pone.0294145)
Supplement: Attachment — Submitted filename: comments to manuscript PONE-D-23-13935-revised.docx [file pone.0294145.s004.docx]

Congyu Jin

# Comments to PONE-D-23-13935 – revised

The authors have responded to the comments by providing new data and making modifications to improve the weakness of the manuscript that has been pointed out. The interaction of age, gender and TERT promoter mutations was not explored in this study due to the limitation of this cohort of patients, it is now mentioned in the discussion.

There are still some mistakes/flaws in the revised manuscript; some of them can be corrected easily:

1. Due to the small sample size of each age and gender subgroup, advanced analysis of age- and gender-specific mortality rates could not be applied. I could not find the “additional Fig 1 and 2” as indicated in the authors’ response. This limitation can be addressed in discussion.

2. To make it clear for readers, “age” could be changed to “age of onset/diagnosis”.

3. The authors have added subtitles to graphs, which makes the manuscript easier to read. Still, in the figure legends, all abbreviations should be illustrated.

4. Figure 3, what is “No. at risk”? Please use the original words instead of abbreviation, and indicate “age of onset/diagnosis) above the age group could be more illustrative.

Age of onset/diagnosis

I think that after the corrections, and also take other reviewers’ opinion into account; this manuscript could be considered to be published on PLOS ONE.
