# Supplementary material for: Age-associated mortality is partially mediated by TERT promoter mutation status in differentiated thyroid carcinoma
Source: PLoS One. 2023 Nov 10;18(11):e0294145. doi: 10.1371/journal.pone.0294145 (PMC10637683; doi:10.1371/journal.pone.0294145)
Supplement: Attachment — Submitted filename: Response to reviewers.docx [file pone.0294145.s005.docx]

**[Response to Reviewers’ comments]**

**Manuscript Number: PONE-D-23-13935R1**

**Article Type: Original article**

**Title: Age-associated mortality is partially mediated by TERT promoter mutation status in differentiated thyroid carcinoma**

**[Journal Requirements]**

Any changes to the reference list should be mentioned in the rebuttal letter that accompanies your revised manuscript.

**🡪 Response**: A new article is now included in the references as reference number 26 to provide an explanation for the abbreviation ‘AAD’ (age at diagnosis). Apart from this addition, there have been no changes to the previous references or reference order.

**[Comments to PONE-D-23-13935 – revised]**

1. Due to the small sample size of each age and gender subgroup, advanced analysis of age- and gender-specific mortality rates could not be applied. I could not find the “additional Fig 1 and 2” as indicated in the authors’ response. This limitation can be addressed in discussion.

**🡪 Response**: Thank you for the thoughtful suggestions of the reviewer. The additional Fig 1 and 2 are as follows.

<Additional Fig 1. Male>	<Additional Fig 2. Female>

We have incorporated this result into the limitation section of the manuscript. However, we did not present or cite the additional figures in the result section because this result lacks statistical power due to small sample sizes of each subgroups, so the result is difficult to explain and interpret. (Line 413-419)

2. To make it clear for readers, “age” could be changed to “age of onset/diagnosis”.

**🡪 Response**: Thank you for the detailed feedback that the authors missed. We replaced ‘age’ with ‘age at diagnosis (AAD)’ in the manuscript, and we cited an article (Ref: 26) that used the abbreviation ‘AAD’ for ‘age at diagnosis’. But, we did not replace ‘age’ in compound terms like ‘age-associated mortality’ with AAD. (Line 123-124)

3. The authors have added subtitles to graphs, which makes the manuscript easier to read. Still, in the figure legends, all abbreviations should be illustrated.

**🡪 Response**: We thank you for pointing out the part the authors overlooked. We have fully illustrated the abbreviations which remained in the figure legends previously.

4. Figure 3, what is “No. at risk”? Please use the original words instead of abbreviation, and indicate “age of onset/diagnosis) above the age group could be more illustrative.

**🡪 Response**: We denoted the original term ‘Number at risk’ in Fig 3. Additionally, we added vertical columns named ‘age at diagnosis’ to the left of the number of AAD groups.
